# Synergistic impacts of low-carbon city pilot policy and information consumption pilot policy on carbon lock-in

**DOI:** 10.3389/fpubh.2026.1929422

**Published:** 2026-10-09

**Authors:** Yan Peng, Rongguang Zhang

**Affiliations:** 1 School of Digital Economy, Sichuan University Jinjiang College, Meishan, China; 2 College of Management Science, Chengdu University of Technology, Chengdu, China

**Keywords:** carbon lock-in, dual machine learning, information consumption pilot, low-carbon city pilot, policy synergy

## Abstract

**Introduction:**

Carbon lock-in poses a significant barrier to the low-carbon transition, necessitating synergistic policy interventions.

**Methods:**

Using a dual machine learning (DML) model, this study estimates carbon lock-in data for 262 Chinese cities and examines how the dual policy (the low-carbon city pilot and information consumption pilot policy) affects carbon lock-in.

**Results:**

The dual policy significantly suppresses carbon lock-in, with a markedly stronger effect than implementing either policy in isolation. The analysis indicates that the dual policy works through industrial structure upgrading, government environmental support, climate technology innovation, and public climate concern. Additionally, the heterogeneity test reveals that the suppressive effect is more pronounced in non-resource-based cities, large cities, and those with high market vitality and strong financial regulation.

**Discussion:**

Consequently, this study provides evidence for promoting the synergistic implementation of the low-carbon city pilot and the information consumption pilot policy, thereby breaking carbon lock-in.

## 1 Introduction

Climate change poses serious threats to public health and economic development, and it is a shared challenge facing human society (1, 2). There is a global consensus on advancing low-carbon transformation to address climate change (3, 4). As a responsible country, China has actively assumed its responsibilities for reducing emissions and has set the dual carbon goals of peaking carbon emissions and achieving carbon neutrality. Nevertheless, the current global emissions reduction landscape remains challenging. The International Energy Agency data reveal that in 2024, global carbon emissions from energy use stood at approximately 37.8 billion tons. China accounted for an astonishing one-third of this total, or 12.6 billion tons. While China’s emissions growth slowed in 2024, its per capita emissions were still 16% higher than those of developed countries, indicating significant pressure to reduce emissions. The challenge is a reflection of China’s contemporary development context. China’s continued progression through industrialization sustains fossil fuel dependence as a persistent structural feature of its economy in the immediate future. Its development pathway is entrenched in a high-carbon energy system, thereby perpetuating a carbon lock-in (5, 6) that severely impedes its low-carbon transition (7, 8).

Carbon lock-in describes a process whereby technological lock-in and institutional path dependence form a technology-institutional complex (TIC) due to the path-dependent and self-reinforcing characteristics of carbon-based technological systems (9, 10). This complex has long dominated economies and societies, locking them into fossil fuel-based energy systems and hindering the adoption and diffusion of low-carbon technologies (11, 12). While breaking carbon lock-in and shifting from conventional energy are essential to addressing climate challenges (10, 13), facilitating this transition remains a paramount challenge shared by China and the international community. Scholars advocate for the integration of market mechanisms with institutional frameworks through policy interventions (6, 14, 15), technological innovation (13, 16), industrial upgrading, and shifts in public behavior (5, 12, 17, 18). This multifaceted approach aims to transition the energy system from a high-carbon trajectory to a low-carbon path, thereby accelerating the overall carbon unlocking process.

In an effort to address climate change, China has launched a series of pilot policies (19, 20). The low-carbon city pilot policy (LCCP) and the information consumption pilot policy (ICPP) are two representative policy initiatives. The LCCP aims to boost energy conservation and cut emissions, thereby advancing broader green and low-carbon growth (19, 21). Meanwhile, the ICPP establishes a new consumption model by merging information technology with conventional consumption practices (22, 23). Existing literature confirms the role of LCCP in reducing emissions and achieving carbon neutrality (12, 24). Additionally, ongoing urbanization has driven an inelastic increase in household energy demand, and public behavioral habits have led to a more pronounced carbon lock-in phenomenon (18). Guiding residential consumption transformation is critical for reducing carbon emissions (25, 26). As an emerging new consumption model, the ICPP can effectively reduce carbon emissions by altering consumption concepts and promoting industrial upgrading (22, 26).

Although individual policies, such as LCCP and ICPP, have significant potential to curb carbon emissions and achieve a low-carbon transformation (12, 22, 26, 27), the existing literature still exhibits research gaps. First, the policy effects of dual or multiple pilot programs have recently gained attention in academia (6, 28, 29). However, prior studies have not analyzed the synergistic effects of combining LCCP and ICPP. Additionally, while successful coordination of policy tools can compensate for the shortcomings of individual instruments, counterproductive combinations may lead to policy conflicts (6, 30–32). The extant studies are also divided on whether the effects of multiple policies outperform those of single policies. Additionally, while prior studies have confirmed the potential of policy instruments in mitigating carbon emissions, they have overlooked the more challenging issue of carbon lock-in. To bridge these gaps, our study employs a dual machine learning (DML) model based on constructed carbon lock-in data from 262 Chinese cities between 2008 and 2022. The study examines the impact and mechanisms of dual policy—LCCP and ICPP—on carbon lock-in and investigates whether the dual policy outperforms the single policy. The findings provide empirical support for policymakers seeking to overcome carbon lock-in.

This study makes three key contributions. First, previous studies have predominantly analyzed the effect of individual policy on carbon lock-in. This approach has overlooked the synergistic effects generated by multiple policies with temporal overlap and spatial interdependence. This study broadens the perspective of research on how policy synergy can reduce carbon lock-in. Second, by examining the mutual reinforcement and complementary mechanisms between LCCP and ICPP, the study unpacks how the dual policy influences carbon lock-in through four channels: technological innovation, government support, industrial upgrading, and public awareness. These findings provide a pathway for China to break carbon lock-in. Furthermore, the study analyzes how urban heterogeneity affects the effect of dual policy on carbon lock-in across resource endowments, city scale, market vitality, and financial regulation. These insights offer actionable insights for crafting context-specific policy mixes, enabling governments to orchestrate the co-implementation of LCCP and ICPP for decarbonization. Third, the study employs a DML model. Capitalizing on the capacity of machine learning for high-dimensional, non-parametric forecasting, the adopted model circumvents the dimensionality constraints and functional form misspecification typical of conventional econometric approaches. This enhances the reliability of the conclusions.

## 2 Literature review and hypotheses development

### 2.1 Policy background

China has constructed a wide-ranging set of policies to combat climate change. A key initiative within this framework is the low-carbon pilot scheme, which was methodically rolled out starting with five provinces and eight cities in 2010 to foster diverse low-carbon practices. A subsequent expansion in 2012 designated 29 cities and provinces, followed by a 2017 cohort that added 45 cities, including Nanjing and Chengdu. The program now totals 81 pilot cities and six pilot provinces. These cities are exploring pathways for low-carbon, green development by adjusting industrial structures, and promoting low-carbon technological innovation.

In 2013, China launched its first batch of information consumption pilot projects. The 68 designated zones comprised cities, counties, and districts, including Beijing and Tianjin, aiming to boost demand and cultivate new economic drivers. In 2015, the ministry selected an additional 36 cities, including Baoding and Xuzhou, for the second pilot phase. These cities have actively fostered emerging consumption patterns, such as digital consumption. In doing so, they have become key drivers in empowering traditional industries to upgrade and catalyzing the emergence of new industries and business models.

### 2.2 Literature review

#### 2.2.1 Carbon lock-in

Unruh (9) pioneered the concept of “carbon lock-in,” explaining how industrial economies become entrenched in high-carbon energy systems due to technological path dependence and increasing returns to scale. The concept’s underlying formation logic stems from “technological lock-in.” As carbon-based technologies mature and improve, it becomes more difficult to replace old technologies with new ones. This leads to a deep dependence on traditional technologies (7, 9, 10). “Institutional lock-in” further excludes new energy systems by establishing policy rules aligned with carbon-based frameworks. Together with the technological system, these form a TIC, which endows the lock-in with self-reinforcing characteristics (10, 11, 33). Furthermore, carbon-intensive products and infrastructure influence societal consumption habits and psychological preferences, creating “social lock-in” that solidifies high-carbon development pathways from behavioral and demand perspectives (34). Regarding measurement, the Carbon lock-in index lacks a unified assessment framework. Some scholars measure carbon lock-in through the net carbon flux, calculated as the balance between emissions and sinks (35), while others prefer composite evaluation indicators (5, 6, 15, 36). For instance, Hu and Xu (5) developed a framework that integrates technology, institutions, and social behavior to evaluate carbon lock-in and test the effects of unlocking carbon through new energy pilot policies. Erickson et al. (37) used four criteria—equipment lifespan, carbon emissions, barriers to developing low-carbon technology, and institutional mechanisms—to evaluate carbon lock-in in major energy-consuming assets, such as power generation and buildings.

Carbon lock-in impedes the development and deployment of low-carbon technologies (11), creating a significant barrier to economic decarbonization (7, 8). Research emphasizes the need for external forces to break the self-reinforcing cycle of carbon lock-in (5, 10, 36). Breaking carbon lock-in requires technological and institutional unlocking (10, 11, 33). Technological unlocking involves shifting away from high-carbon technologies and prioritizing energy efficiency improvements and emission reductions as key breakthroughs (8, 13, 36, 38). Examples include enhancing energy efficiency through digitalization (39) and incentivizing the development and widespread deployment of low-carbon technologies (13, 40). Institutional unlocking encourages governments to dismantle existing technology-institutional complexes through policy interventions and institutional innovations (5, 6, 14). This includes leveraging mechanisms such as the carbon trading policies and the LCCP to guide the low-carbon transition (15, 20). Concurrently, demand-side behavioral shifts can be stimulated by implementing carbon taxes or by enhancing the perceived value of low-carbon products among consumers, thereby facilitating societal carbon unlocking (18).

#### 2.2.2 Dual policy

Regarding the environmental effects of the single policy of LCCP and ICPP, existing studies have demonstrated that LCCP plays a significant role in improving energy efficiency (41, 42), reducing carbon emissions (19, 43), promoting green behaviors (44, 45), and improving air quality (46). In terms of transmission mechanisms, this policy relies on command environmental regulation as its core instrument, imposing constraints through emission caps, energy efficiency standards, and industrial access thresholds, which in turn force the exit of high-carbon industries (15). At the same time, it is supplemented by local governments’ environmental investment (19), which induces green technological innovation such as clean energy substitution (12), and it enhances public low-carbon awareness through community outreach (44). Furthermore, with respect to ICPP, existing literature has confirmed that it can reduce urban carbon emissions (22, 26) and is conducive to green development and enhancing economic resilience (23, 47). In terms of transmission mechanisms, this policy takes demand-side pulling as its starting point, promoting industrial structure upgrading through e-commerce and digital services (26), relying on government public investment in digital infrastructure (23) to foster digital technologies such as artificial intelligence and big data for efficient resource allocation (48), and stimulating technological innovation (49), while simultaneously reshaping residents’ consumption habits through digital media and strengthening consumers’ environmental awareness (22).

Recently, scholars have begun to study the synergistic effects of combining macro policies (6, 31, 50, 51). A policy combination is a common approach to correcting market failures, and successful synergies between policy tools can compensate for the shortcomings of individual instruments (6, 51). For instance, Zhang et al. (29) examined the impact of combining low-carbon, innovation, and new urbanization pilot programs on low-carbon green development. They found that the effects of three policies were most pronounced, while the effects of two policies from different sets were diminished compared to single policies. Shen et al. (6) noted that the dual implementation of new energy and digital policies has significant synergistic effects on carbon emission reduction, with combination policies demonstrating more significant enabling effects than standalone measures. However, such combinations are not universally effective. Without effective coordination, conflicts and negative effects may arise among tools (32, 52).

Although extant literature provides a reference for our study, certain research gaps remain. First, although the environmental effects of single policies have been extensively discussed, and both LCCP and ICPP can generate positive environmental effects through the four pathways of industry, government, technology, and society. There are notable differences in their respective focal points and logics of action: the former emphasizes regulatory constraints and green technology traction on the production side, whereas the latter emphasizes digital reshaping and efficiency empowerment on the consumption side. However, existing literature has largely focused on the pathway analysis of single policies, lacking a systematic analysis of the pathway differences between the two types of policies within the same analytical framework. Second, there remains disagreement regarding the policy synergy effects of the dual pilots, and whether the dual-pilot policy significantly outperforms single policies requires further empirical testing. These gaps provide the entry point for this study.

### 2.3 The dual policy and carbon lock-in

The LCCP serves as a pilot initiative that advances green and low-carbon transitions, ensuring the achievement of dual carbon objectives. According to existing research, the LCCP reduces carbon emissions and breaks carbon lock-in by fostering green technological innovation, streamlining industrial structures, and cultivating public adoption of environmentally friendly habits (12, 15, 19, 27). Additionally, the ICPP facilitates the advancement of information infrastructure, the creation of intelligent information products, and the establishment of new model initiatives for information consumption. These efforts harness online retail ecosystems and social networks to transition purchasing habits from traditional offline channels towards blended online-offline experiences, thereby reducing pollution and emissions associated with traditional consumption models (53, 54). Furthermore, the pilot program optimizes resource allocation and enhances resource utilization efficiency through digital infrastructure development and digital technology advancement (23), which in turn drives structural progress and technological advancement, ultimately leading to lower carbon emissions (22, 26) and achieving carbon unlocking.

Compared to single pilot cities, the dual policy shows significant synergistic and complementary effects in breaking carbon lock-in (55). These effects primarily manifest in three ways: alignment of strategic objectives, coordinated flow of resources, and collaborative transformation of consumption patterns. First, the dual pilot policy exhibits consistency in strategic goals. The LCCP is designed to achieve emission cuts by spearheading the development of a low-carbon ecosystem in energy and industry (19, 56). Meanwhile, the ICPP drives industrial transformation and consumption upgrading by promoting information consumption and developing the digital economy (26, 57). This achieves high-quality development. Both policies are committed to advancing the economic system from traditional, extensive models toward innovation-driven, green, and low-carbon approaches. This high degree of alignment at the objective level lays the foundation for policy synergy. Second, the dual pilot policy optimizes the allocation of production factors, generating a multiplier effect through coordinated resource flows. The LCCP guides capital, talent, and other factors to withdraw from high-emission sectors and flow toward green industries through green industry orientation and policy constraints (58). The ICPP stimulates data vitality as a factor by promoting the more efficient matching and sharing of traditional factors, such as capital, technology, and talent, through digital platforms (23). This synergy breaks down barriers to factor mobility and jointly constructs a new system for allocating factors in the green innovation economy, amplifying the combined impact of policies. Finally, the dual policy drives the transformation of consumption patterns toward greener, more digital practices. The LCCP encourages green consumption by promoting low-carbon lifestyles, green buildings, and transportation, as well as developing low-carbon industries (44, 45). Meanwhile, the ICPP has profoundly altered people’s lifestyles and consumption habits by developing new sectors, such as digital content, smart home technology, and the mobile internet. This has facilitated the embrace of pro-environmental lifestyles, exemplified by the rise of telecommuting, digital entertainment, and the sharing economy (48). Together, these policies integrate and reinforce low-carbon concepts and digital models, driving consumption pattern transformation and creating a favorable social foundation for overcoming the carbon lock-in dilemma.

Furthermore, the dual policy’s complementary mechanism primarily manifests in three ways: the complementary effectiveness of policy instruments, the complementary supply and demand of technological development, and the complementary structures of governance. First, the dual pilot policy achieves complementary effectiveness in policy instruments. As an environmental regulatory tool, the LCCP enforces strict limitations through emission targets and energy efficiency standards (42, 43). In contrast, the ICPP emphasizes market incentives, such as tax incentives, market-based investment and financing encouragement, and business model innovation support, to stimulate market vitality (23). Second, the dual policy fosters complementary supply and demand dynamics in technological development. By establishing clear emission reduction goals and environmental regulations, the LCCP creates a reliable market pull for low-carbon technologies. Meanwhile, the ICPP enhances technological supply capacity by strengthening infrastructure development and research and development support (23, 26). Third, the dual policy demonstrates structural complementarity in governance models. With its focus on top-down planning, the LCCP obliges cities to undertake the formulation of comprehensive low-carbon strategies and the establishment of stringent oversight mechanisms, reflecting strong government leadership and macro-level coordination. The ICPP, however, places greater emphasis on stimulating grassroots vitality and diverse participation, encouraging local governments, enterprises, research institutions, and consumers to jointly explore new technologies, new business formats, and new models. This complementary governance structure collectively shapes a new collaborative governance paradigm characterized by “government guidance, market leadership, enterprise participation, and social involvement,” thereby enhancing governance effectiveness.

Building on the preceding analysis, our study advances hypothesis 1 (H1): The dual policy is more effective than a single pilot policy in breaking carbon lock-in.

### 2.4 Mechanisms

#### 2.4.1 Industrial structure upgrading

The dual policy drives industrial restructuring through policy guidance and market mechanisms. The LCCP compels enterprises to innovate technologically through environmental regulations and carbon emission constraints (27, 43). This reduces reliance on traditional, high-carbon industries and redirects resources toward emerging sectors. Thus, it optimizes the industrial structure (12, 15). The LCCP promotes optimizing energy structures, increasing clean energy usage, and improving overall energy efficiency (41, 42). This provides foundational support for industrial upgrading. Furthermore, the ICPP fosters new business models and formats by expanding digitalization in consumption sectors. This stimulates demand-side transformation and drives industries toward service-oriented and knowledge-based transitions (22, 23). By improving information infrastructure, these pilot policies provide foundational support for industrial digitization. Integrating digital technologies with traditional industries accelerates their modernization, enhancing production efficiency and intelligence (26, 48).

The industrial sector is the major contributor to carbon emissions (59). Traditional high-carbon industries, such as heavy manufacturing and fossil fuel dependency, have rigid structures that lead to carbon lock-in. Upgrading industrial structures can optimize resource allocation and reduce dependence on high-carbon pathways by driving the economy’s transition from high-carbon to low-carbon models, such as developing services, renewable energy, and green manufacturing (20). This directly breaks industrial-level lock-in effects and promotes sustainable development. Therefore, our study identifies industrial structure upgrading as the core element of industrial unlocking pathways and proposes hypothesis 2a (H2a): The dual policy breaks carbon lock-in by driving industrial structure upgrading.

#### 2.4.2 Government environmental support

Government regulation is a powerful tool for addressing market failures and environmental issues (42, 60). The dual pilot policy enhances government support for green and low-carbon transformations through strategic design. Traditional environmental regulations often rely on singular, mandatory, command-and-control approaches that can impose high compliance costs on businesses (60, 61). In contrast, the LCCP gives local governments more flexibility and autonomy. It encourages local governments to develop low-carbon industry plans based on local industrial structures and resource endowments while strengthening supporting measures, such as green finance. This creates a policy combination characterized by “weak constraints and strong incentives.” Furthermore, the ICPP explicitly calls for “increasing financial support to advance R&D in cutting-edge information consumption technologies,” which directly addresses the core challenges of high upfront investments in digital technologies and widespread corporate funding shortages. Together, these two pilot programs significantly reduce the initial costs and risks of low-carbon technology and digital transformation by providing direct financial support, such as fiscal subsidies and green credit. This approach greatly enhances the precision and effectiveness of policy interventions.

Government support, reinforced by the dual policy, is the key to breaking institutional lock-in (36, 62). Institutional carbon lock-in often stems from policy inertia, market failures, and regulatory gaps (7). By implementing supportive measures such as environmental regulations, carbon pricing, subsidies, and public investment, governments can create incentive frameworks that address externalities and direct businesses and the public toward low-carbon transitions. This process helps overcome path dependence. Consequently, this study identifies government environmental support as the core element for institutional unlocking and proposes hypothesis 2b (H2b): The dual policy breaks carbon lock-in by promoting government environmental support.

#### 2.4.3 Climate technology innovation

Technological resources are essential for enterprises to drive technological innovation (63). The LCCP prioritizes the development and deployment of clean technologies, including new energy solutions and carbon capture systems. Meanwhile, the ICPP facilitates the widespread integration of digital technologies, leveraging tools such as AI and cloud computing. The dual policy jointly establishes the resource foundation supporting climate technology innovation. The introduction of digital technologies effectively breaks down information barriers, enabling enterprises to access critical climate technology innovation information and providing foundational support for knowledge transfer and collaborative research and development (R&D) (64). Concurrently, low-carbon city development attracts highly skilled talent by improving environmental quality and offering ecological benefits. Additionally, growth in information consumption generates numerous technology-intensive positions, further driving human capital concentration in pilot regions. This concentration reduces talent acquisition costs for enterprises, thereby accelerating climate technology innovation (48).

Technology lock-in is characterized by the dominance of traditional, high-carbon technologies and insufficient innovation (40). Climate technology innovation provides viable alternatives to traditional technologies by promoting and applying low-carbon solutions, such as renewable energy and carbon capture. Furthermore, by reducing emission costs, accelerating technology diffusion, and overcoming technical bottlenecks, climate technology innovation drives industrial and societal transformation (65), which is essential for breaking the carbon lock-in. Thus, this study regards climate technology innovation as a core element of the technology unlocking pathway and proposes hypothesis 2c (H2c): The dual policy breaks carbon lock-in by promoting climate technology innovation.

#### 2.4.4 Public climate concern

The dual pilot policy establishes an integrated online and offline system for disseminating information that significantly increases public awareness of climate issues. The LCCP requires the “vigorous promotion of public education and outreach activities, encouragement of low-carbon lifestyles and behaviors, and advancement of low-carbon living.” This demonstrates the government’s and all sectors of society’s strong commitment to low-carbon development, fostering a shared consensus on low-carbon development among the public. Furthermore, the ICPP accelerates the rapid development of e-commerce and online consumption. The growing prevalence of smartphones and smart devices in daily life has significantly improved the efficiency and accessibility of disseminating information. This facilitates public access to climate-related knowledge and information, thereby enhancing consumer climate concern (26).

Social carbon lock-in stems from habits, consumption patterns, and culture (34). Public concern about climate change spurs bottom-up change by raising awareness, altering lifestyles, and generating social pressure. This spurs individual actions, such as energy-efficient consumption and support for environmental policies, thereby breaking societal inertia. Therefore, this study identifies public climate concern as the key element for unlocking social behavior and proposes hypothesis 2d (H2d): The dual policy breaks carbon lock-in by promoting public climate concern.

The framework illustrating how the dual policy influences carbon lock-in is presented in Figure 1.

**Figure 1:**
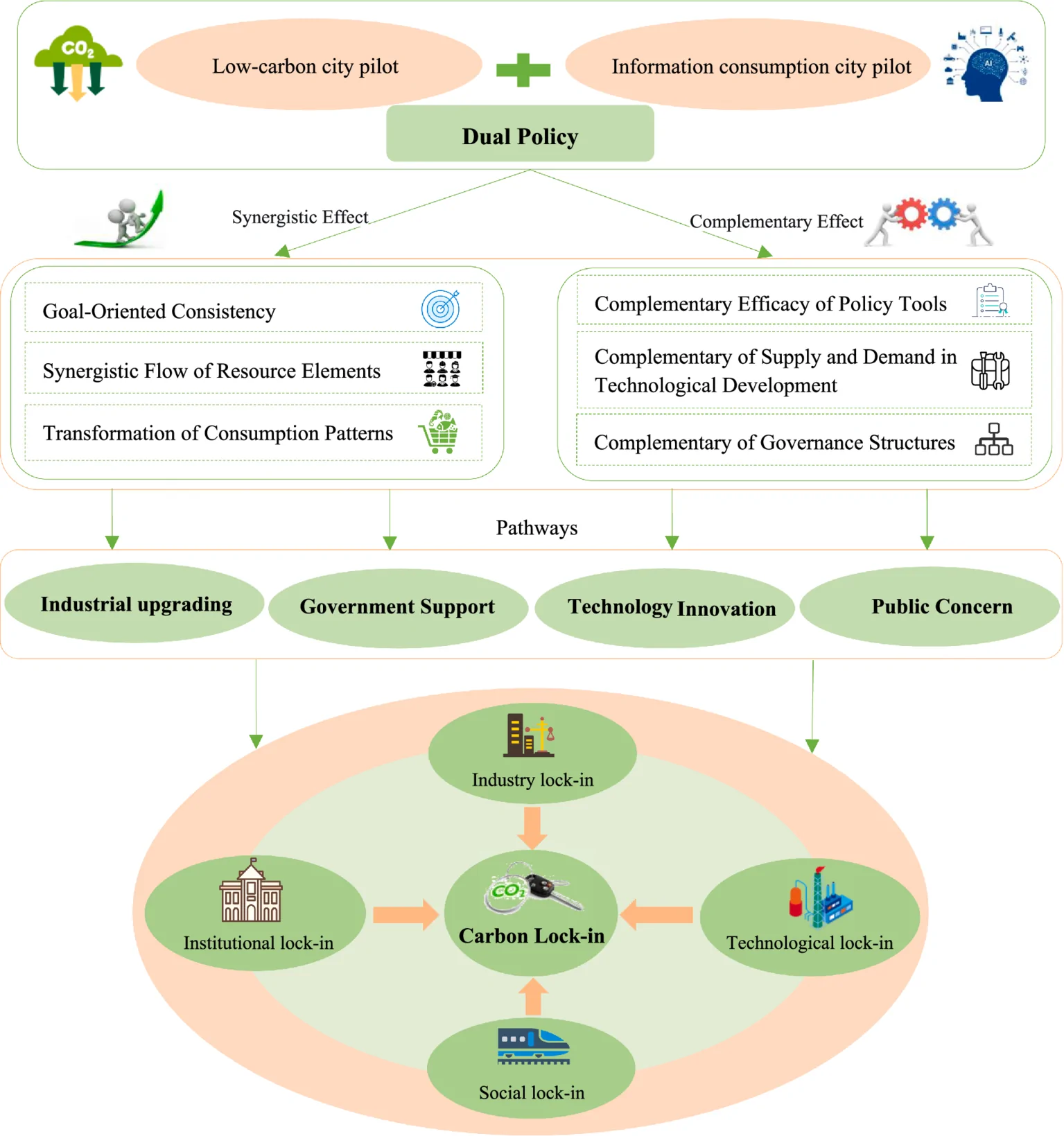
The logical framework of dual policy influencing carbon lock-in.

## 3 Materials and methods

### 3.1 Model

To address the constraints inherent in traditional causal inference frameworks for policy assessment, this study draws upon Shen et al. (6) and Hu and Xu (5) to construct the following partial linear DML and auxiliary regression model for analyzing the role of dual policy in mitigating carbon lock-in.


$$\mathrm{CBL}K_{\mathrm{it}}=\theta_{0}\text{Polic}y_{\mathrm{it}}+g(X_{i})+\varepsilon_{\mathrm{it}}E(\varepsilon_{\mathrm{it}}|,\text{Polic}y_{\mathrm{it}}|,X_{i})=0$$ (1)

$$\text{Polic}y_{\mathrm{it}}=m(X_{\mathrm{it}})+v_{\mathrm{it}}E(v_{\mathrm{it}}|X_{\mathrm{it}})=0$$ (2)

Here, *i* and *t* denote city and year, respectively; $\mathrm{CBL}K_{\mathrm{it}}\;$ represents the city carbon lock-in; $\text{Polic}y_{\mathrm{it}}\;$ is the dual policy; $\theta_{0}\;$ is the estimated coefficient; $X_{i}\;$ denotes a set of high-dimensional control variables, each exerting influence through $m(X_{\mathrm{it}})$ and $g(X_{i})$ , whose specific forms require estimation via machine learning algorithms. Finally, $\varepsilon_{\mathrm{it}}$ and $v_{\mathrm{it}}$ represent error terms.

### 3.2 Variables

#### 3.2.1 Carbon lock-in

Referring to Liu et al. (65), Zhao et al. (66), Zhou and Qi (20), this study constructs carbon lock-in indicators across industrial, institutional, technological, and social dimensions. At the industrial level, the contribution of secondary industry in regional GDP is employed to reflect the proportion of high-energy-consumption, high-emission industries within the overall economic system (65). Furthermore, our study uses the number of high-carbon firms to measure the agglomeration scale and structural rigidity of high-emission industries at the micro level, reflecting the entrenched nature of carbon-intensive production patterns and the pressure for low-carbon transition (67). The registered capital of climate-friendly firms is selected to gauge the potential for industrial structure transformation toward low-carbon development (68, 69). At the institutional level, the number of climate policies and the severity of climate penalty reflect the government’s emphasis on energy conservation, emission reduction, and environmental governance (70). Furthermore, the share of science and technology expenditures in fiscal spending is used to gauge government investment in carbon unlocking (12). At the technological level, this study uses indicators of carbon and energy intensity to evaluate the performance of low-carbon technologies in practice, the capabilities of regional emission reduction, and future potential (20, 66). Additionally, we use R&D expenditure as an indicator of the allocation of urban resources to low-carbon technologies (65). Since human capital is a vital resource for technological innovation (71, 72), we assess urban technological innovation capacity using the number of R&D staff. At the social level, urban population density reflects the intensity of social activities within cities. Dense residential patterns significantly increase resident interaction frequency and mobility demands, thereby elevating energy consumption (20). This study adopts the mathematical definition of urban population density as population per unit area. Furthermore, heightened environmental awareness among residents can gradually shift preferences from high-carbon to low-carbon lifestyles. The quantity of public transportation systems in a city indicates public preference for low-carbon travel modes (38).

The construction of the specific indicators is detailed in Table 1. For positive indicators, we normalize by (X−min(X))/(max(X)−min(X)) and for negative indicators, we normalize by (max(X)−X)/(max(X)−min(X)) . Subsequently, we use the CRITIC objective assignment method to generate the indicator weights for the *i-*th indicator (Equations 3, 4):

$$w_{i}=\frac{C_{i}}{\sum_{j}^{n}C_{j}},i=1,2\ldots ,n$$ (3)

$$C_{i}=\sigma_{i}\sum_{j}^{n}(1-r_{\mathrm{ij}}),i=1,2\ldots ,n,i\neq j$$ (4)

where $\sigma_{i}$ is the standard deviation of indicator *i,* and $r_{\mathrm{ij}}$ is the correlation coefficient between indicator*s i* and indicator *j*. Indicators *i* and *j* are subordinate indicators that have the same indicator.

**Table 1:** Carbon Lock-in Indicators.

Indicator	Measurement	Property
Industry lock-in	The share of the added value of secondary industry in GDP	+
	The share of high-carbon firms in the total number of firms	+
	Climate-friendly firm registered capital	−
Institution lock-in	The proportion of technology expenditures in fiscal expenditures	−
	Climate policy	−
	Climate penalty	−
Technology lock-in	R&D staff	−
	Ratio of R&D expenditures to GDP	−
	Ratio of energy consumption to GDP	+
	Ratio of carbon emission to GDP	+
Social behavior lock-in	Population density	+
	Number of public transportation vehicles	−

Then, the carbon lock-in index is incremented by one, and its natural logarithm is taken to derive *CBLK* as a proxy variable for urban carbon lock-in. Figure 2 presents the distribution of the carbon lock-in index. The figure reveals a declining trend in China’s carbon lock-in from 2008 to 2022. However, the spatial imbalances and regional concentration patterns underlying this trend precisely reflect the current distribution of energy consumption and carbon emissions.

**Figure 2:**
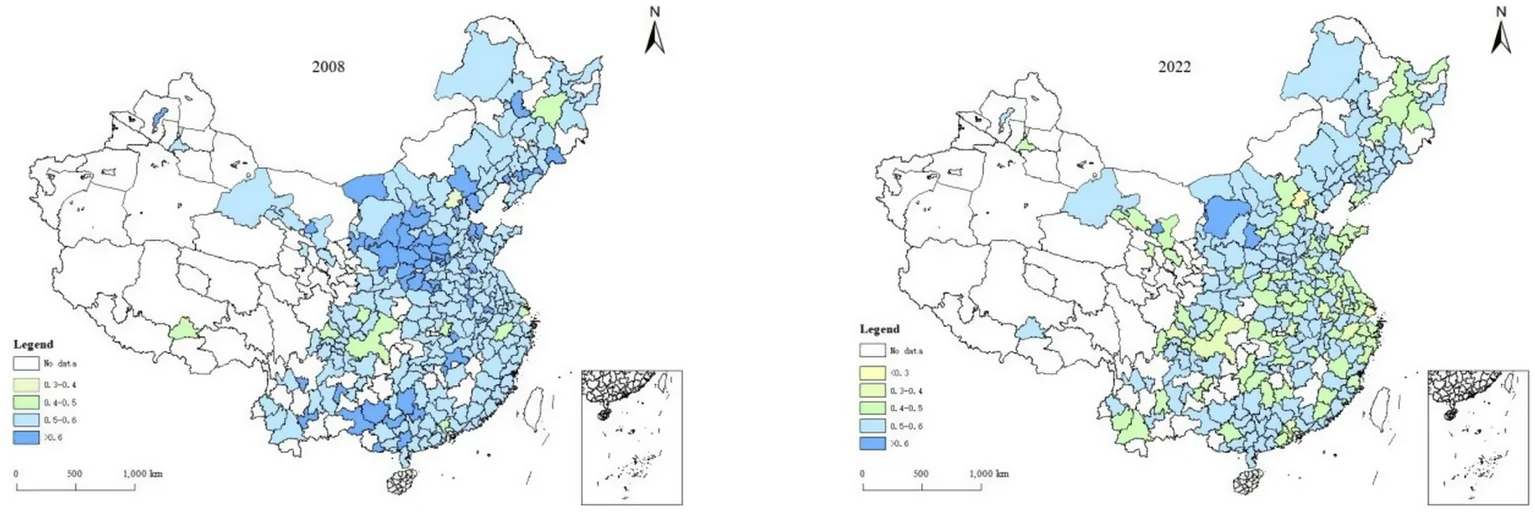
Distribution map of carbon lock-in in Chinese cities.

#### 3.2.2 Dual policy

The dual policy takes a value of 1 if a city qualifies as both a low-carbon and an information consumption pilot in the initial year or later years, and 0 if not. Similarly, for the LCCP or the ICPP, the pilot city is set to 1 if the city is designated in the same year or later years; otherwise, it is 0. Counties with severe data missingness are excluded. Within the sample, there are 74 single low-carbon pilot cities, 34 single information consumption pilot cities, 45 dual pilot cities, and 109 non-pilot cities. The specific distribution is shown in Figure 3.

**Figure 3:**
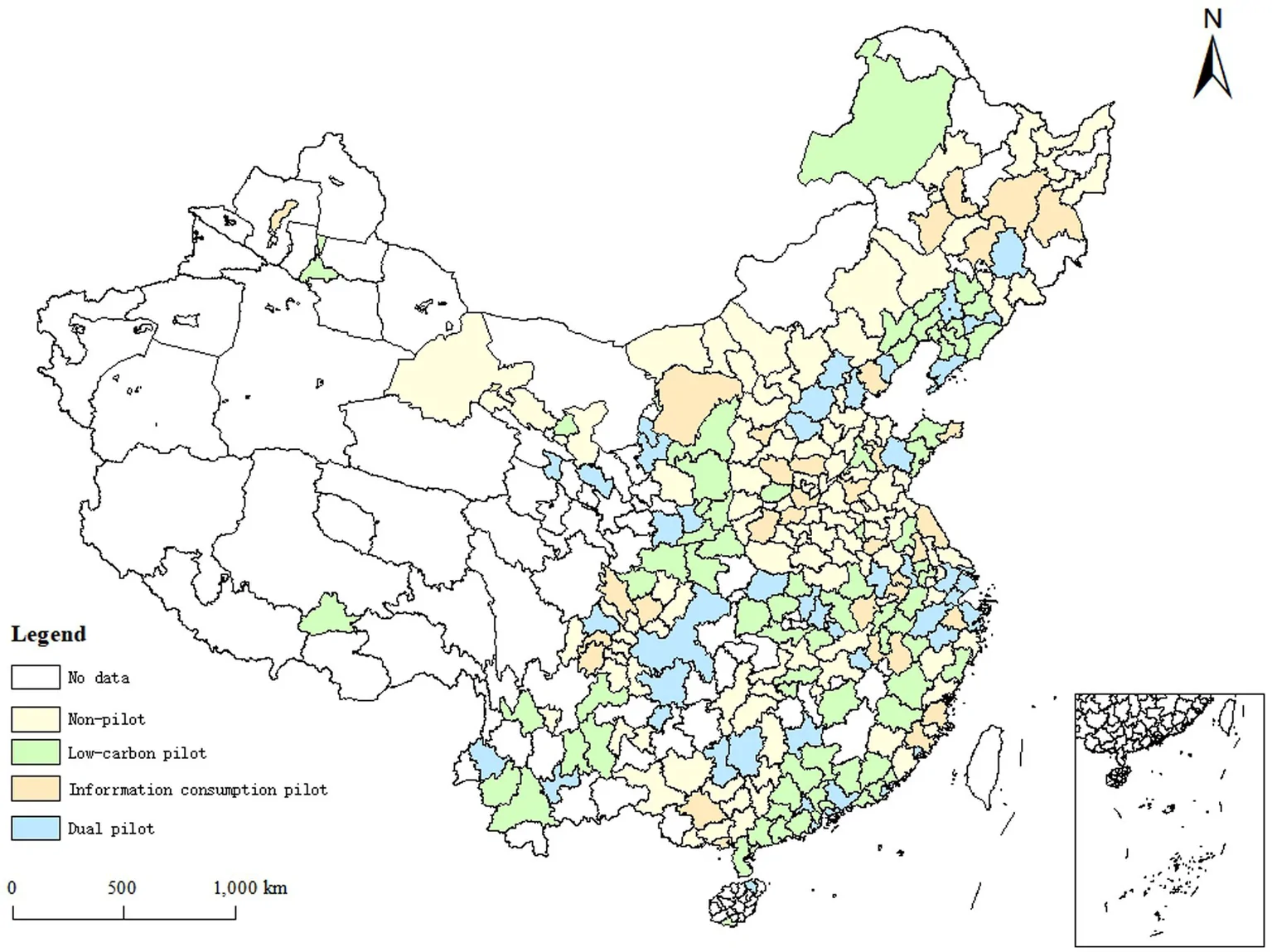
The spatial distribution of pilot cities.

#### 3.2.3 Control variables

Following the method of Zhao et al. (36), Liu et al. (65), Zhou and Qi (20), we control for the following variables: Economic growth (*GGDP*) is measured by the real GDP growth rate. Financial development (*FSIZ*) is calculated as total deposits and loans as a percentage of GDP. Financial efficiency (*FEFF*) is calculated as the loan-to-deposit ratio. Human capital level (*HCAP*) is quantified by the share of college students in the city’s total population. Urbanization rate (*CITY*) is calculated as the proportion of the population residing in urban districts relative to the total population.

### 3.3 Data

Data on high-carbon firms and the registered capital of climate-friendly firms are sourced from the Enterprise Warning Platform^1^. This study identifies keywords from foundational policy texts, including the “Climate Finance Pilot Notice” and the “State Council Climate Work Conference Communication.” Based on these keywords, the study collects data on the cumulative count of climate policies issued annually by each city from the Bailu Think Tank. This study compiles the yearly number of penalties against firms in each city from Qichacha’s environmental records as a measure of climate penalties, using “atmospheric regulations” and “air pollution” as keywords. The carbon emissions are obtained from the CEADs (China Emission Accounts and Datasets) database. City-level emissions are derived from county-level emissions data; the most recent county-level data are from 2017. For emissions from 2018 to 2022, provincial-level emissions are obtained from the CSMAR database and weighted by GDP to derive city-level emissions. Additional data sources include the China City Statistical Yearbook, China Energy Statistical Yearbook, the China Science and Technology Statistical Yearbook, and statistical bulletins.

In constructing the carbon lock-in indicators, cities with extensive data gaps are excluded, and remaining missing values are imputed via linear interpolation. This process yields a final sample size of 262 cities. In 2008, China released “China’s Policy and Action on Climate Change (2008),” marking the beginning of its emphasis on and implementation of multiple carbon reduction policies. Therefore, this study uses panel data from 262 prefecture-level Chinese cities between 2008 and 2022 to analyze the effect of dual policy on carbon lock-in. All variables except those in logarithmic form are winsorized at the 1th and 99th percentiles to minimize outlier effects. This process yields a final sample of 3,823 city-year observations. The descriptive statistics of the primary variables are displayed in Table 2.

**Table 2:** Descriptive statistics of key variables.

Variables	Observations	Mean	Std. dev.	Min	Max
*CBLK*	3,823	0.4296	0.0361	0.0909	0.5561
*GGDP*	3,823	0.0888	0.0437	−0.0330	0.2008
*FSIZ*	3,823	2.4813	1.2680	0.6240	10.5729
*FEFF*	3,823	0.6810	0.1933	0.2874	1.2654
*INDS*	3,823	2.2941	0.1428	1.9003	2.6734
*HCAP*	3,823	1.8456	2.3947	0.0000	11.4709
*CITY*	3,823	0.3732	0.2376	0.0619	1.0000

## 4 Results

### 4.1 Benchmark regression

Our study uses the DML model specified in Equations 1, 2 to test the effects of the dual policy on carbon lock-in. We employ the random forest algorithm for both main and auxiliary regressions using a 1:4 sample split, with the outcomes reported in Table 3. Columns (1)–(4) show the results of the dual policy with and without control variables and their quadratic terms. The findings reveal that the estimated coefficients are significantly negative at the 1% level, demonstrating the effectiveness of the dual policy in reducing carbon lock-in. Subsequently, we report tests with the LCCP and ICPP as key explanatory variables, presenting estimates for these models in columns (5) and (6). Both single pilot policies also exhibit carbon lock-in reduction effects. However, the dual policy’s coefficient (0.0075) is significantly greater in magnitude than those of the LCCP (0.0052) and ICPP (0.004). This demonstrates that the impact of the dual policy exceeds that of a single policy. Furthermore, when we treat dual-pilot and non-pilot cities as the treatment sample (excluding those that implement only one policy), the results in column (7) show that, after controlling for single-policy interference, the dual policy still suppresses carbon lock-in significantly. Finally, we test the net policy effect of dual pilots relative to single pilots after excluding non-pilot cities (i.e., all samples are cities implementing at least one pilot policy). As presented in column (8), the significantly negative coefficient demonstrates that the dual policy has a more pronounced effect than a single policy in inhibiting carbon lock-in. In other words, the joint implementation of two policies has a stronger policy impact through synergistic effects than a single policy. Therefore, H1 is validated.

**Table 3:** Results of the benchmark regression.

Variables	*CBLK*							
	(1)	(2)	(3)	(4)	(5)	(6)	(7)	(8)
Dual policy	−0.0962***	−0.0073***	−0.0080***	−0.0075***				
	(0.0040)	(0.0024)	(0.0024)	(0.0024)				
LCCP					−0.0052***			
					(0.0012)			
ICPP						−0.0040***		
						(0.0016)		
Dual and no							−0.0114***	
							(0.0023)	
Dual and single								−0.0085***
								(0.0024)
Control	No	Yes	No	Yes	Yes	Yes	Yes	Yes
Control^2	No	No	Yes	Yes	Yes	Yes	Yes	Yes
City FE	Yes	Yes	Yes	Yes	Yes	Yes	Yes	Yes
Year FE	Yes	Yes	Yes	Yes	Yes	Yes	Yes	Yes
Observations	3,823	3,823	3,823	3,823	3,823	3,823	2,214	2,268

Robust standard errors in parentheses; ***, **, and * are significant at the level of 1, 5, and 10% respectively, the same as in the following tables.

Further comparing the effects of the two single pilot policies, the absolute coefficient of LCCP (0.0052) is slightly larger than that of ICPP (0.0040). This difference may be related to the policy attributes: the LCCP is an environmental regulatory policy that directly acts on the emission side through administrative constraints such as carbon intensity targets and industrial structure adjustment (15, 43). Whereas the ICPP is a market-incentive policy that indirectly promotes the green transformation of economic activities by improving digital infrastructure and cultivating new consumption patterns (22, 26). The former has a more direct effect, while the latter focuses more on generating long-term momentum for low-carbon transformation from the demand and consumption side.

### 4.2 Robustness tests

#### 4.2.1 Test for heterogeneity in treatment effects

Given that the pilot policies in this study were implemented at different times, using a traditional two-way fixed-effects model to examine the economic impact of the exogenous shock of distance changes may result in estimation biases due to heterogeneous treatment effects. To address this issue, this study first decomposes the estimation results of the multi-period DID, following the approach of Goodman-Bacon (73). Column (1) of Table 4A reports the estimation results. Overall, the coefficient obtained using this method is −0.0085, which is significant at the 5% level, consistent with the results of the baseline regression. Table 4B further reports the decomposition results across different groups. This study finds that the estimation results primarily stem from the treatment group and the group that has never received treatment, accounting for 97.04% of the total. In these groups, treatment effects are generally not subject to heterogeneity caused by time-varying factors.

**Table 4:** Heterogeneity in treatment effects.

(A)		
Variables	(1)	(2)
	Baconcomp	CSDID
Dual policy	−0.0085**	−0.0059**
	(0.0034)	(0.0024)
Observations	3,029	3,820

(B)		
Categories	Coefficient	Weights
Earlier treated vs. Later treated	−0.0047	0.0146
Later treated vs. Earlier treated	−0.0027	0.0150
Treated vs. Never treated	−0.0085	0.9704

Although the Bacon decomposition verifies that the issue of heterogeneous treatment effects is unlikely to be a major concern in this study, we further employ the method proposed by Callaway and Sant’Anna (74), namely the CSDID, to test the robustness of the multi-period DID. The results are reported in Column (2) of Table 4A. We find that the coefficient of the robust estimator is −0.0059 and is statistically significant at the 5% level, which further confirms that estimation bias due to heterogeneous treatment effects does not pose a serious problem in our study.

#### 4.2.2 Multiple cross-validation

Since DML requires multiple cross-validations to mitigate the variance induced by random sample splitting, we repeat the sample-splitting process 50 times, each time randomly partitioning the sample (keeping the split ratio at 1:4), and take the average and standard error of the 50 estimated coefficients as the final results. Column (1) of Table 5 presents the results after this multiple cross-validation, showing that the coefficient of the dual-pilot policy remains significantly negative, indicating that our conclusions are not affected by random sample splitting and further confirming the robustness of the baseline results.

**Table 5:** The results of the robustness test.

Variables	Multiple executions	Change sample	5% Winsorize	Province-time fixed effect	Exclude other policies	
					Smart city	Carbon trade
	(1)	(2)	(3)	(4)	(5)	(6)
Dual policy	−0.0096***	−0.0100***	−0.0052***	−0.0085***	−0.0072***	−0.0045*
	(0.0026)	(0.0023)	(0.0017)	(0.0022)	(0.0023)	(0.0023)
Control	Yes	Yes	Yes	Yes	Yes	Yes
Control^2	Yes	Yes	Yes	Yes	Yes	Yes
City FE	Yes	Yes	Yes	Yes	Yes	Yes
Year FE	Yes	Yes	Yes	Yes	Yes	Yes
Observations	3,823	3,069	3,823	3,823	3,823	3,823

Variables	Adjust the proportion of sample segmentation		Replace DML			Endogenous test
	1:2	1:7	GBM	LassoCV	Interactive model	
	(7)	(8)	(9)	(10)	(11)	(12)
Dual policy	−0.0079***	−0.0088***	−0.0112***	−0.0116***	−0.0130***	−0.0906***
	(0.0023)	(0.0023)	(0.0023)	(0.0023)	(0.0015)	(0.0312)
Control	Yes	Yes	Yes	Yes	Yes	Yes
Control^2	Yes	Yes	Yes	Yes	Yes	Yes
City FE	Yes	Yes	Yes	Yes	Yes	Yes
Year FE	Yes	Yes	Yes	Yes	Yes	Yes
Observations	3,823	3,823	3,823	3,823	3,823	3,823

#### 4.2.3 Changing samples

This study omits the four centrally administered municipalities (Beijing, Tianjin, Shanghai, and Chongqing) due to their distinct administrative status, which often leads to preferential resource allocation and divergent policy processes compared to other cities. The remaining city samples are utilized for regression analysis, with findings reported in column (2) of Table 5, confirming the robustness of the study’s conclusions after the sample adjustment.

#### 4.2.4 Adjusting the winsorization percentage

The regression estimates under this robust treatment of variables (5th/95th percentile winsorization) are presented in Table 5, column (3), confirming that the findings are not driven by extreme values.

#### 4.2.5 Accounting for provincial time interactions

As provinces constitute a pivotal administrative tier in China’s governance system, cities under the same provincial jurisdiction tend to share commonalities in policy frameworks, geographic advantages, and socio-historical contexts. Thus, the baseline regression includes province-time interactive fixed effects to absorb time-varying provincial shocks, with the findings reported in column (4) of Table 5.

#### 4.2.6 Controlling for similar policies

To assess the discrete effect of the policy, the empirical model accounts for other similar initiatives launched concurrently. The carbon trading policy aims to promote green and low-carbon development (20). The smart city pilot program improves urban governance efficiency through information technology (75). We include policy variables for carbon trading and smart cities in the regression, with the findings shown in columns (5)–(6) of Table 5.

#### 4.2.7 Resetting machine learning models

To mitigate the effect of bias in DML models on conclusions, our study adjusts the sample split ratio from 1:4 to 1:2 and 1:7. Furthermore, we use Gradient Boosting Model (GBM) and Lasso Cross Validation (LassoCV) to examine the potential effect on the conclusions. Additionally, we use the DML to construct an interactive model that mitigates the subjectivity inherent in benchmark regression settings. The results after model reconfiguration are presented in columns (7)–(11) of Table 5 and confirm that the study’s conclusions remain unaffected by DML.

#### 4.2.8 Endogeneity treatment

To address endogeneity issues in the model, this study employs Bartik instrumental variables, based on the method of Borusyak et al. (76) and Shen et al. (6). Specifically, this study constructs the instrumental variable by interacting the number of fixed-line telephones per 100 people in 1984 with the growth rate of the national climate policy. The historical number of phones per 100 people reflects the initial endowment differences in information infrastructure across regions, and such differences directly determine the capacity foundation for subsequently undertaking the pilot policy. Furthermore, the national climate policy growth rate is a macro-level policy variable that is externally determined by national-level decision-making and independent of unobserved error terms at the city level. Column (12) of Table 5 reports the results after endogeneity treatment, and the regression coefficient remains significantly negative.

### 4.3 Mechanism test

Referring to Zhang et al. (77), we utilize a dual mediation analysis to investigate the transmission mechanisms through which the dual policy influences carbon lock-in. As detailed in Table 6, the analysis demonstrates that the dual policy alleviates urban carbon lock-in through four pathways: industrial structure upgrading, government environmental support, climate technology innovation, and public climate awareness. The effects of these four pathways, ranked by magnitude, are as follows: technological innovation effect (0.1788% ≈ 0.0112 × 0.1596), government support effect (0.1428% ≈ 0.3571 × 0.004), industrial upgrading effect (0.1225% ≈ 0.0174 × 0.0704), and public concern effect (0.0862% ≈ 0.6632 × 0.0013).

**Table 6:** The results of mechanism testing.

Variables	Industrial upgrading		Government expenditures		Technological Innovation		Public Concern	
	*INDS*	*CBLK*	*GOEN*	*CBLK*	*CFTI*	*CBLK*	*CLCN*	*CBLK*
	(1)	(2)	(3)	(4)	(5)	(6)	(7)	(8)
Dual policy	0.0174***		0.3571***		0.0112***		0.6632***	
	(0.0061)		(0.0769)		(0.0026)		(0.1498)	
*INDS*		−0.0704***						
		(0.0052)						
*GOEN*				−0.0040***				
				(0.0005)				
*CFTI*						−0.1596***		
						(0.0239)		
*CLCN*								−0.0013***
								(0.0002)
Control	Yes	Yes	Yes	Yes	Yes	Yes	Yes	Yes
Control^2	Yes	Yes	Yes	Yes	Yes	Yes	Yes	Yes
City FE	Yes	Yes	Yes	Yes	Yes	Yes	Yes	Yes
Year FE	Yes	Yes	Yes	Yes	Yes	Yes	Yes	Yes
Observations	3,823	3,823	3,823	3,823	3,823	3,823	3,823	3,823

#### 4.3.1 Channels for industrial structure upgrading

To test the effectiveness of industrial structure upgrading, we use the formula (1 × primary industry value-added + 2 × secondary industry value-added + 3 × tertiary industry value-added) to get industrial structure upgrading (*INDS*) (38), then conduct a regression analysis. The estimates in columns (1) and (2) of Table 6 summarize the dual policy’s effect on industrial upgrading and the latter’s impact on carbon lock-in, respectively. A significantly positive coefficient for the policy and a negative one for industrial upgrading confirm that the dual policy mitigates carbon lock-in by facilitating industrial structure upgrading, thereby supporting H2a.

#### 4.3.2 Channels for government environmental support

To test the influence of the dual policy on urban carbon lock-in through government environmental support, we calculate government environmental support (*GOEN*) as the natural logarithm of government environmental spending plus one. Columns (3) and (4) in Table 6 present the impact of the dual policy on government environmental support and the effect of government environmental support on carbon lock-in, respectively. The results indicate that the coefficient for the dual policy is significantly positive, while the coefficient for government environmental support is significantly negative. These results confirm that the dual policy significantly reduces urban carbon lock-in by promoting government environmental support, thus validating H2b.

#### 4.3.3 Channels for climate technology innovation

To investigate how the dual policy influences urban carbon lock-in through climate technology innovation, this study manually collected patent application counts for CPC classification Y02 (defined as technologies mitigating or adapting to climate change) from the Wisdom Tooth. The per capita climate-friendly patent application count was calculated to derive *CFTI* as a proxy variable for climate technology innovation (68). The analysis in columns (5) and (6) of Table 6 assesses the dual policy’s positive influence on climate technology innovation and the latter’s suppressive effect on carbon lock-in. The significantly positive and negative coefficients, respectively, confirm that the policy reduces carbon lock-in by fostering climate innovation, thereby validating H2c.

#### 4.3.4 Channels for public climate concern

To explore how the dual policy influences urban carbon lock-in through public climate concern, we extract keywords from related documents, including the Guiding Opinions on Promoting Climate Change Financing and Investment and the China Climate Change Policy and Action Report 2022. Using the format “city + keyword,” we obtain word frequency counts from China Daily to measure public climate concern (*CLCN*) and conduct a regression analysis. As reported in columns (7) and (8) of Table 6, the dual policy significantly boosts public climate concern, which itself exerts a significant negative influence on carbon lock-in. The positive and negative coefficients, respectively, confirm that elevating public concern serves as an effective channel for the policy to alleviate carbon lock-in, thus validating H2d.

### 4.4 Heterogeneity analysis

#### 4.4.1 Resource endowment

Given the variation in resource availability among cities, we classify the sample into resource-based (REC) and non-resource-based (Non-REC) cities, following the National Sustainable Development Plan for Resource-Based Cities. A grouped regression approach is employed to test the differential roles of the dual policy on carbon lock-in. As reported in columns (1) and (2) of Table 7, the estimated effect of the dual policy is insignificant in REC cities (coefficient = −0.0046), whereas it is significantly negative at the 1% level in Non-REC cities (coefficient = −0.0124). These results reveal that the dual policy effectively reduces carbon lock-in in the Non-REC cities but is less effective in the REC cities. This may be because the REC cities’ extensive production models rely heavily on carbon-intensive industries. Upgrading and transforming these industries is costly and faces significant resistance, which subjects them to greater short-term economic pressures and technological bottlenecks. Constrained by path dependence and insufficient innovation resources, the policy’s effects are relatively weaker in these cities. Conversely, the economic activity of Non-REC cities is concentrated in industries characterized by minimal emissions, low consumption, and independence from natural resources (5). This enables them to promote the absorption and transformation of clean technologies flexibly and efficiently, facilitating the transition of energy and industrial structures.

**Table 7:** The results of heterogeneity analysis.

Variables	Resource endowment		City scale		Market vitality		Financial Regulation	
	Rec	Non	SMC	LC	Low	High	Low	High
	(1)	(2)	(3)	(4)	(5)	(6)	(7)	(8)
Dual policy	−0.0046	−0.0124***	−0.0082***	−0.0106***	−0.0051	−0.0158***	−0.0050*	−0.0147***
	(0.0055)	(0.0024)	(0.0032)	(0.0029)	(0.0038)	(0.0027)	(0.0028)	(0.0053)
Control	Yes	Yes	Yes	Yes	Yes	Yes	Yes	Yes
Control^2	Yes	Yes	Yes	Yes	Yes	Yes	Yes	Yes
City FE	Yes	Yes	Yes	Yes	Yes	Yes	Yes	Yes
Year FE	Yes	Yes	Yes	Yes	Yes	Yes	Yes	Yes
Observations	1,525	2,298	1,342	2,481	1,916	1,907	2,900	923

#### 4.4.2 City scale

Since city scale significantly impacts policy implementation outcomes, this study uses population as the metric for city scale. Based on the 2014 State Council criteria for city scale classification, cities are categorized as large cities (LC) if their population surpasses three million, and as small and medium-sized cities (SMC) if it falls below. As presented in columns (3) and (4) of Table 7, the dual policy exerts a significant negative influence on carbon lock-in in both small and medium-sized cities (coefficient = −0.0082) and large cities (coefficient = −0.0106), with all estimates significant at the 1% level. This confirms the policy’s universal effectiveness across the urban hierarchy. Notably, the policy’s suppression effect on carbon lock-in is more pronounced in large cities. This may be because small cities are lagging in economic development and have weaker economic foundations, which makes implementing pilot programs more challenging. Large cities, in contrast, possess abundant research resources, digital infrastructure, and human capital, granting them greater advantages in economies of scale and technological innovation (38).

#### 4.4.3 Market vitality

Market vitality is a key driver of innovation (78). To account for its impact, this study adopts Chen and Zhang’s (68) methodology, using enterprise density, defined as the number of firms per unit area, to capture market vitality. The sample cities are stratified into low and high market vitality groups, applying a median split based on the sample’s own distribution. As reported in columns (5) and (6) of Table 7, the dual policy has no significant effect in cities with low market vitality. Conversely, in cities with high market vitality, the dual policy significantly inhibits carbon lock-in. This may be because individuals in cities with high market vitality more readily establish social networks and engage in frequent interactions. These conditions facilitate the dissemination and accumulation of knowledge, thereby promoting climate technology innovation (68, 78).

#### 4.4.4 Financial regulation

Substantial financial investment is required to achieve carbon unlocking (65). Financial regulation plays a key role in determining the allocation of financial resources (79) and may therefore influence the process of unlocking carbon lock-in. To analyze the impact of financial regulation, we collect data on the number of penalties imposed by the China Banking and Insurance Regulatory Commission on banks and their branches within cities through the Enterprise Warning Platform, which serves as a measure of financial regulatory intensity. We stratify cities into low- and high-intensity regulatory groups at the sample median of the financial regulation. As reported in columns (7) and (8) of Table 7, the coefficients for the dual policy are significantly negative in both types of cities. Furthermore, the policy has a greater mitigating effect on carbon lock-in in cities with high financial regulatory intensity. This may be because financial regulation standardizes bank lending practices and enhances internal control quality (80), which corrects non-compliant lending and ineffective green credit policy implementation. This redirects credit resources toward real enterprises, alleviating corporate financing constraints and boosting their ability for technological innovation and investment in green projects (81).

## 5 Discussion

Breaking carbon lock-in is essential for addressing climate change and achieving low-carbon transition (5, 10, 12). This paper examines the impact of the dual policy (LCCP and ICPP) on carbon lock-in. Our findings are consistent with existing literature in some aspects, yet they also differ in others.

First, at the theoretical framework level of carbon lock-in, this study verifies the applicability of the TIC theory proposed by Unruh (9, 10) in the context of Chinese cities. The study finds that the dual policy can reduce carbon lock-in through four major paths: industrial structure upgrading, government environmental support, climate technology innovation, and public climate concern. This is highly consistent with the “industry-institution-technology-society” four-dimensional structure of the carbon lock-in formation mechanism (20, 65, 66), indicating that breaking carbon lock-in requires a multi-pronged and systematic approach. Second, at the level of single policy effect, this study once again confirms the positive role of LCCP in carbon emission reduction and carbon unlocking (12, 19, 24), and also provides evidence for the positive environmental effects of ICPP (22, 23, 26). More importantly, this study confirms the effectiveness of policy synergy, a conclusion consistent with relevant literature on policy synergy research (6, 28, 29, 77). This thus responds to the academic attention to policy synergy effects in recent years.

Compared to existing research, this study also achieves differentiated expansion. First, from a research perspective, previous literature on carbon unlocking has mostly focused on single policy tools, such as low-carbon pilots (12), carbon trading markets (20), or new energy policies (5), but rarely incorporated environmental regulation-based policies and consumption promotion-based policies into a unified analytical framework. This paper systematically compares the synergistic effects of two types of heterogeneous policies, namely LCCP and ICPP, on carbon lock-in for the first time, revealing their mutual promotion mechanisms in terms of goal consistency, factor flow, and consumption transformation, as well as their complementary logic in terms of policy instruments, technology supply and demand, and governance structures. This expands the theoretical boundaries of policy synergy research. Furthermore, existing literature mostly discusses the heterogeneity of urban characteristics and geographical locations (12, 36, 38, 65). This paper supplements the existing heterogeneity analysis of carbon unlocking research from the perspectives of market vitality and financial regulation. It reveals the boundary conditions for policy effects to be exerted, providing a reference for local governments to optimize policies according to local conditions and break carbon lock-in.

## 6 Conclusion

This study employs panel data on estimated carbon lock-in for 262 Chinese cities from 2008 to 2022. Using the DML model with a random forest algorithm, it investigates the effect of the dual policy on carbon lock-in. The analysis further examines the robustness, underlying mechanisms, and heterogeneity of these effects. The findings reveal that: First, the dual policy has significantly suppressed carbon lock-in. Compared with non-pilot cities, dual-pilot cities have achieved an average reduction of 0.75% in carbon lock-in. The finding is robust to a battery of robustness checks, including sample adjustments, outlier removal, controlling for similar policies, incorporating time-province interactions, switching DML models, and addressing endogeneity. Furthermore, the dual policy exhibits a significantly stronger suppressive effect than a single policy, underscoring the value of policy coordination. Second, mechanism analysis identifies four transmission channels: industrial structure upgrading, government environmental support, climate technology innovation, and public climate concern. Third, heterogeneity analysis shows that the policy’s suppressive effect is more pronounced in non-resource-based cities, large cities, and those with high market vitality and strong financial regulation.

Drawing on the empirical results, this study advances the following policy implications. First, governments should strengthen the coordinated implementation of low-carbon and information consumption pilot programs by establishing collaborative mechanisms from the outset of policy design. At the national level, unified guidelines should be established to coordinate the dual pilot programs, explicitly requiring pilot cities to integrate objectives such as reducing carbon intensity and increasing the share of the digital economy when formulating implementation plans. A cross-departmental joint review and advancement mechanism should also be established. Regarding policy tools, pilot regions should be encouraged to bundle policies such as information consumption vouchers and digital infrastructure subsidies with low-carbon incentives, such as energy-saving technology upgrades and green product certifications.

Second, to systematically mitigate carbon lock-in effects, the government should focus on four key dimensions: industrial structure, government support, technological innovation, and public awareness. The government should prioritize the development of an industrial ecosystem that deeply integrates digitalization and environmental sustainability. Using data from information consumption pilot programs and market demand, capital and resources should be directed toward emerging low-carbon industries. The government should establish dedicated green digital industry development funds and provide digital carbon finance tools to offer robust institutional incentives and risk-sharing mechanisms for corporate technological innovation. To promote public participation, the government should leverage information dissemination channels to popularize knowledge about low-carbon practices and promote digital applications for calculating personal carbon footprints.

Third, the government should implement differentiated strategies based on urban heterogeneity and prioritize policy support for non-resource cities, large cities, and regions with market and financial advantages. Policies should prioritize building a green finance ecosystem that balances development and security. While encouraging financial innovation, comprehensive oversight must be strengthened to effectively prevent and mitigate financial risks during the transition.

This study analyzes the role of dual policy in mitigating carbon lock-in, with a focus on Chinese cities. However, several limitations remain. First, the measurement indicators used in this study are primarily based on existing research. The selection of relevant variables and the assignment of weights involve a degree of subjectivity. Constructing such a comprehensive index inevitably introduces non-classical measurement errors, which stem from indicator selection, data availability, and weighting methods. As more granular data becomes available, future research should optimize the indicator system to more objectively characterize the extent of carbon lock-in and the effectiveness of policy interventions. Second, given that China operates under a state-led economic model (68), the findings’ applicability to market-driven economies is unclear. Future work should conduct a comparative analysis of cross-country differences in economic structures, institutional frameworks, and cultural contexts. Third, while the study demonstrates the policy’s mitigating effect on urban carbon lock-in, it does not examine its potential for spatial spillover. Future research should explore the associated spatial interaction effects and cross-regional linkage mechanisms.

## Data Availability

The original contributions presented in the study are included in the article/Supplementary material, further inquiries can be directed to the corresponding author/s.

## Footnotes

1
www.qyyjt.cn
